# Creation of a quality assurance and Regulatory Specialist role: A pilot study

**DOI:** 10.1016/j.conctc.2026.101690

**Published:** 2026-09-21

**Authors:** Elizabeth E. Robison, Devon M. Estes, Jaskaran S. Halait, Katelyn L. Trigg

**Affiliations:** University of California, Davis, Medical Center, Infectious Diseases Research Unit, United States

## Abstract

Quality assurance activities and research compliance activities are paramount to a healthy and successful research program to ensure participant safety and data integrity are upheld. The UC Davis Infectious Diseases Research Unit (IDRU) supports both interventional and observational research, and about half our portfolio consist of studies that are observational. This article will discuss our experience in creating a unique Quality Assurance and Regulatory Specialist role and how it impacted the quality of research in our unit.

## 1 Background

The monitoring process ensures human research subject protections and verifies study data integrity, while also ensuring that the study site is compliant with external and internal regulations. Within our unit, most of our observational studies are investigator-initiated; as a Sponsor-Investigator, the Principal Investigator (PI) takes on the responsibilities of a Sponsor, including monitoring the study data. Among industry sponsored research and interventional studies, monitoring is typically tasked by the sponsor to review this data through a third party, Contract Research Organization (CRO). However, our site set out to instead create an in-house Quality Assurance role embedded within a Regulatory Specialist position.

The University of California, Davis (UC Davis) is a large academic institution that operates within the larger University of California (UC) system. As a clinical research site in California, USA, our research is governed by regulatory requirements at the international, federal, state, and institutional levels. Internationally, all clinical research is conducted according to the International Council for Harmonisation Good Clinical Practice (ICH GCP) guidelines. Federal oversight is provided by the Food and Drug Administration (FDA), while ethical oversight is provided institutionally through Institutional Review Boards (IRB). California also mandates additional research protection laws [1]. And lastly, the UC system and UC Davis maintain policies, independent review committees, and operational procedures which outline best practices to uphold these regulatory requirements.

At UC Davis clinical research units are independently managed by their associated Department and are not centrally managed. While allowing for more autonomy from the Department in conducting research, this structure can also be limiting to smaller Departments or Departments trying to develop a clinical research portfolio.

The Infectious Diseases Research Unit (IDRU) was established 13 years ago and has undergone expansive changes since COVID-19 as the Unit exponentially grew but also discovered many processes and internal practices required a higher level of oversight. The IDRU historically supported clinical research coordinators that completed all clinical study management duties, regulatory duties, startup, and supportive ancillary tasks.

As we found ourselves in a peri-pandemic time our Unit determined the necessity to create a role that could allow for more flexibility, insight into areas of improvement and training opportunities, and a higher level of oversight of our data and quality processes.

### 1.1 Study auditing activities

As part of this pilot, auditing activities were established by a core team of specialists within the IDRU, the Regulatory and Quality Assurance team (RQA). The RQA team created a communication scheme to ensure that activities were covering the IDRU needs, including ensuring data integrity and tracking quality findings. As such, this process was iterative and utilized much of a Process Improvement framework for its success.

In the first iteration of the activities, we established which domains were key in monitoring quality.

### 1.2 Creation of an audit toolkit

After iteration two, a toolkit was developed by the RQA team. The toolkit was inspired by the Quality Assurance Checklist utilized by the Division of Microbiology and Infectious Diseases (DMID) within the National Institute of Allergy and Infectious Diseases (NIAID) [2].

After several iterations and internal discussions, we expanded on the checklist to create a toolkit that included a conditionally formatted spreadsheet that included key domains of interest for our quality team. The toolkit consists of 10 domains: Informed Consent (ICF), Eligibility, Source Documentation (Source), Safety, Specimen collection (Laboratory), EDC Queries (EDC), Other/Misc Subject (Misc), Investigator, and Regulatory.

The inclusion of an Investigator toolkit was discussed and implemented to streamline the resolution of findings. The Investigator aspects of the toolkit include their documentation of study eligibility, complete and accurate source for their assessments (such as physical exam or disease assessments), safety monitoring (including the assessment and documentation of abnormal labs, adverse events, and severe adverse events), and EDC query resolution (if any open queries require the Investigator's sign off).

Ultimately, we decided to utilize colored codes to more easily convey the deadlines per finding. This was decided with our clinical coordinator team through focus groups as part of the pilot. Red fields are the most important as they include GCP or Sponsor compliance findings, addressed immediately (Safety, ICF, and Eligibility). Orange fields consist of internal compliance findings, addressed within 5 business days (Source, EDC). And lastly, yellow fields are for miscellaneous findings, to be addressed within 10 business days (Laboratory, Misc, Investigator, and Regulatory).

### 1.3 Regulatory duties

Prior to the pilot, clinical research coordinators were a jack of all trades coordinator with a breadth of knowledge on all aspects of clinical trial and research management, rather than a depth of knowledge [3]. Following the creation of a QARS role, and in addition to carving out quality assurance duties, this role took on regulatory duties from the existing clinical research coordinators.

The regulatory expectation was to support all aspects of regulatory submissions on behalf of the study team. A handoff occurs at SIV that transfers the regulatory responsibility to the QARS from the startup team. Post-SIV, the QARS will edit consent documents, submit IRB reports, review and triage safety reporting, and manage the regulatory binder.

## 2 Communication

The internal findings are communicated to the study team via DocuSign, where the clinical coordinator or Investigator receives a copy of the filled audit toolkit which details any action items that the QARS identified and their deadline(s). The receiving party must fill in the date they resolved each finding and sign off on the completion of all findings in the report. It is the QARS’ responsibility to follow up on outstanding action items if past due and to support the coordinator and investigator in their resolution, if applicable. This may include an email reminder, or a phone call/virtual/in-person meeting to discuss the findings and create and implement a CAPA.

## 3 Methods

To evaluate whether the separation of duties and creation of a QARS role improved the quality of study conduct and data, we evaluated the sponsored monitoring visit findings pre- and- post-implementation. We evaluated 7 studies’ sponsored monitoring reports. Three studies were conducted before the silo and are considered our baseline, and the other four studies were conducted post-silo and are considered our experimental group. We characterized and quantified all sponsored monitoring visit findings into the 10 domains that we use internally.

In an effort to increase the generalizability of this pilot study, we decided to group study findings by trial type, e.g., treatment or prevention. This was chosen to compare the silo's effect on different study types. We used the Wilcoxon Rank Sum test to compare the sum of pre-silo studies' sponsored monitoring findings to the sum of post-silo studies' sponsored monitoring findings. We also generated box plots of each category of findings pre and post silo which yielded Q1, Q3, and median values.

## 4 Results

Table 1 shows the raw categorical data across the 7 studies, grouped into prevention and treatment trials before and after the silo. Fig. 1, Fig. 2 are visual representations of Table 1.

**Table 1:** CATEGORICAL data, grouped by treatment or prevention study, pre VS post silo.

	ICF	Eligibility	Source	Safety	Laboratory	EDC	Misc	Regulatory	PD	Investigator
**Presilo-prevention**	53	0	0	1	4	2	1	69	63	34
**Presilo-treatment**	0	3	7	0	0	1	1	15	2	0
**Postsilo-treatment**	0	0	1	0	1	1	0	0	3	0
**Postsilo-prevention**	3	0	2	0	0	0	1	3	16	4

**Fig. 1:**
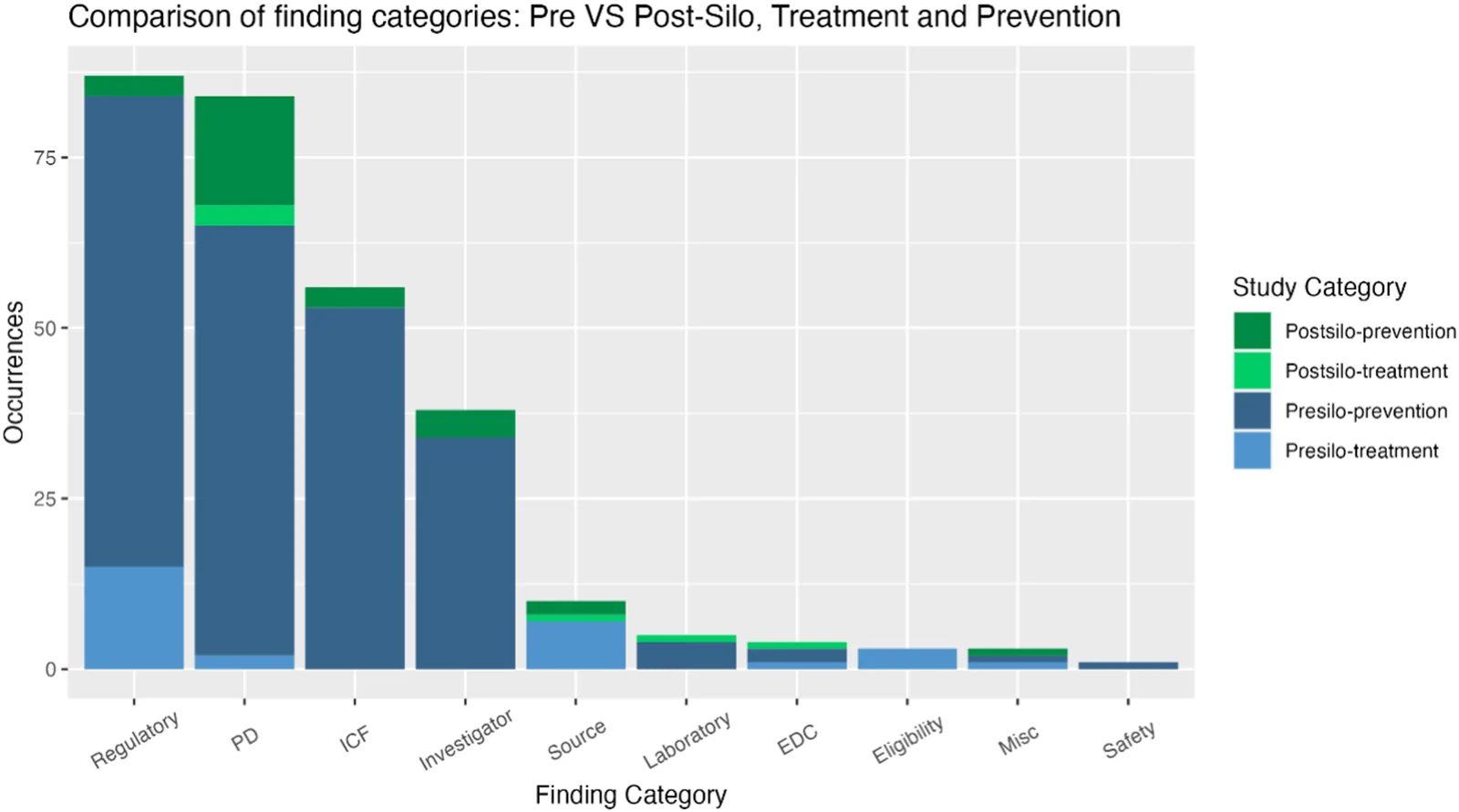
“Comparison of finding categories: Pre VS Post-Silo, Treatment and Prevention”.

**Fig. 2:**
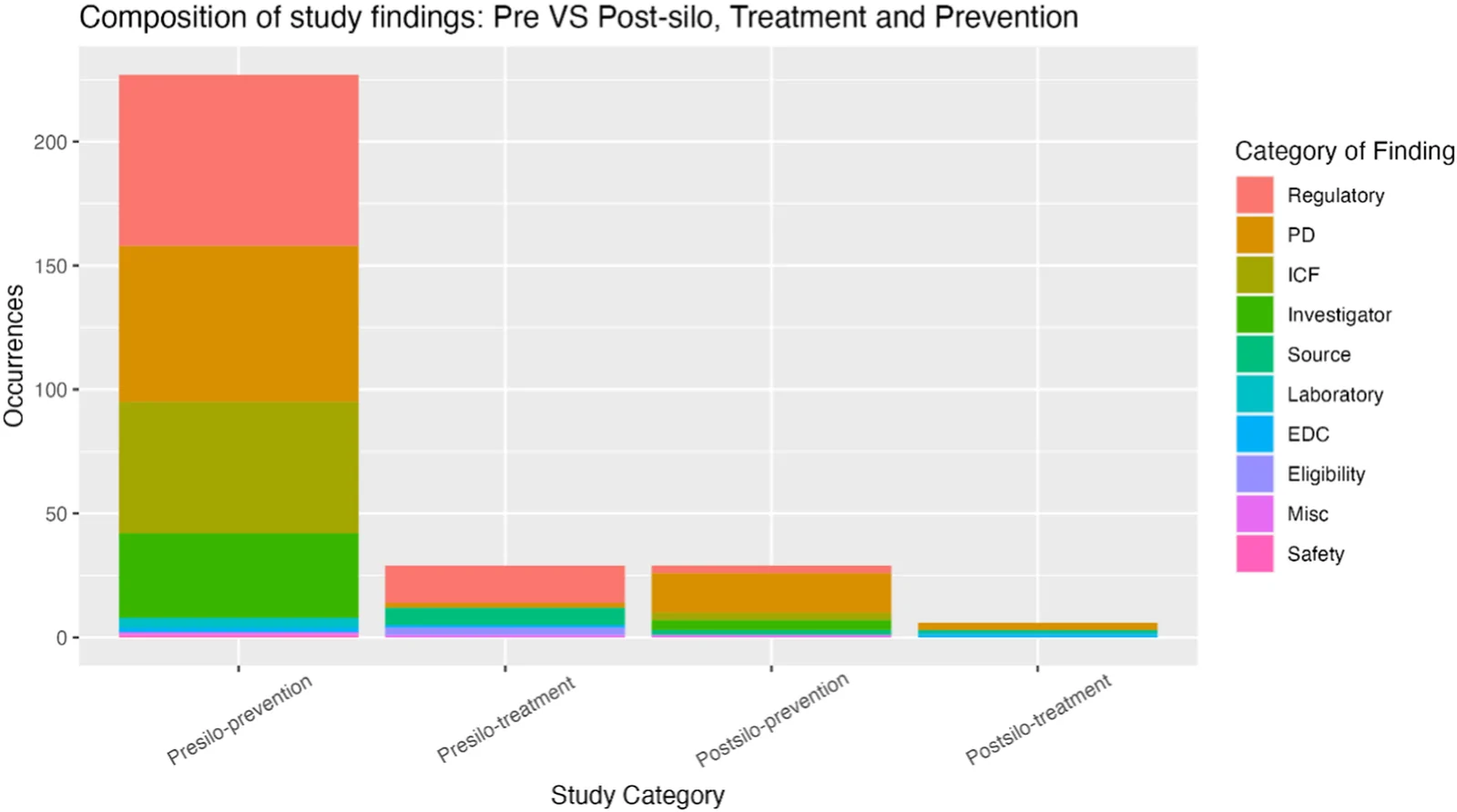
“Composition of study findings: Pre VS Post-silo, Treatment and Prevention”.

Table 2 shows the pre and post silo sum of findings per category, and the results of the Wilcoxon Rank Sum test. Using the Wilcoxon Signed Rank Test Critical Values Table [4], at α = 0.005, we can reject the null hypothesis. We have sufficient evidence to say with 99.5% confidence that the number of findings pre and post silo is not equal.

**Table 2:** Wilcoxon Rank sum, categorical data sum, pre VS post silo.

	Pre-Silo Sum	Post-Silo Sum	Differences	Absolute Differences	Rank	Positive Rank	Negative Rank
ICF	53	3	50	50	9	9	0
Eligibility	3	0	3	3	4.5	4.5	0
Source	7	3	4	4	6	6	0
Safety	1	0	1	1	1.5	1.5	0
Laboratory	4	1	3	3	4.5	4.5	0
EDC	3	1	2	2	3	3	0
Misc	2	1	1	1	1.5	1.5	0
Regulatory	84	3	81	81	10	10	0
PD	65	19	46	46	8	8	0
Investigator	34	4	30	30	7	7	0
Results:							
Smaller Sum				0			
Sample Size				10			

Table 3 shows the results of the boxplots Q1, median (Q2), and Q3 values of the pre and post silo findings per category. Fig. 3 is a visual representation of the boxplot data. When comparing the interquartile ranges, After the silo there is a statistically significant reduction in the total number of findings, and Q3 values, across all categories [5].

**Table 3:** Box plot Q1, Q2, and Q3 values, pre and post silo.

	Pre-Silo Q1	Pre-Silo Q2	Pre-Silo Q3	Post-Silo Q1	Post-Silo Q2	Post-Silo Q3
ICF	0	0	26.5	0	0	0.75
Eligibility	0	0	1.5	0	0	0
Source	0	0	3.5	0	0.5	1.25
Safety	0	0	0.5	0	0	0
Laboratory	0	0	2	0	0	0.25
EDC	0.5	1	1.5	0	0	0.25
Misc	0.5	1	1	0	0	0.25
Regulatory	7.5	12	40.5	0	0	0.75
PD	1	1	32	0.75	2	6
Investigator	0	0	17	0	0.5	1.5

**Fig. 3:**
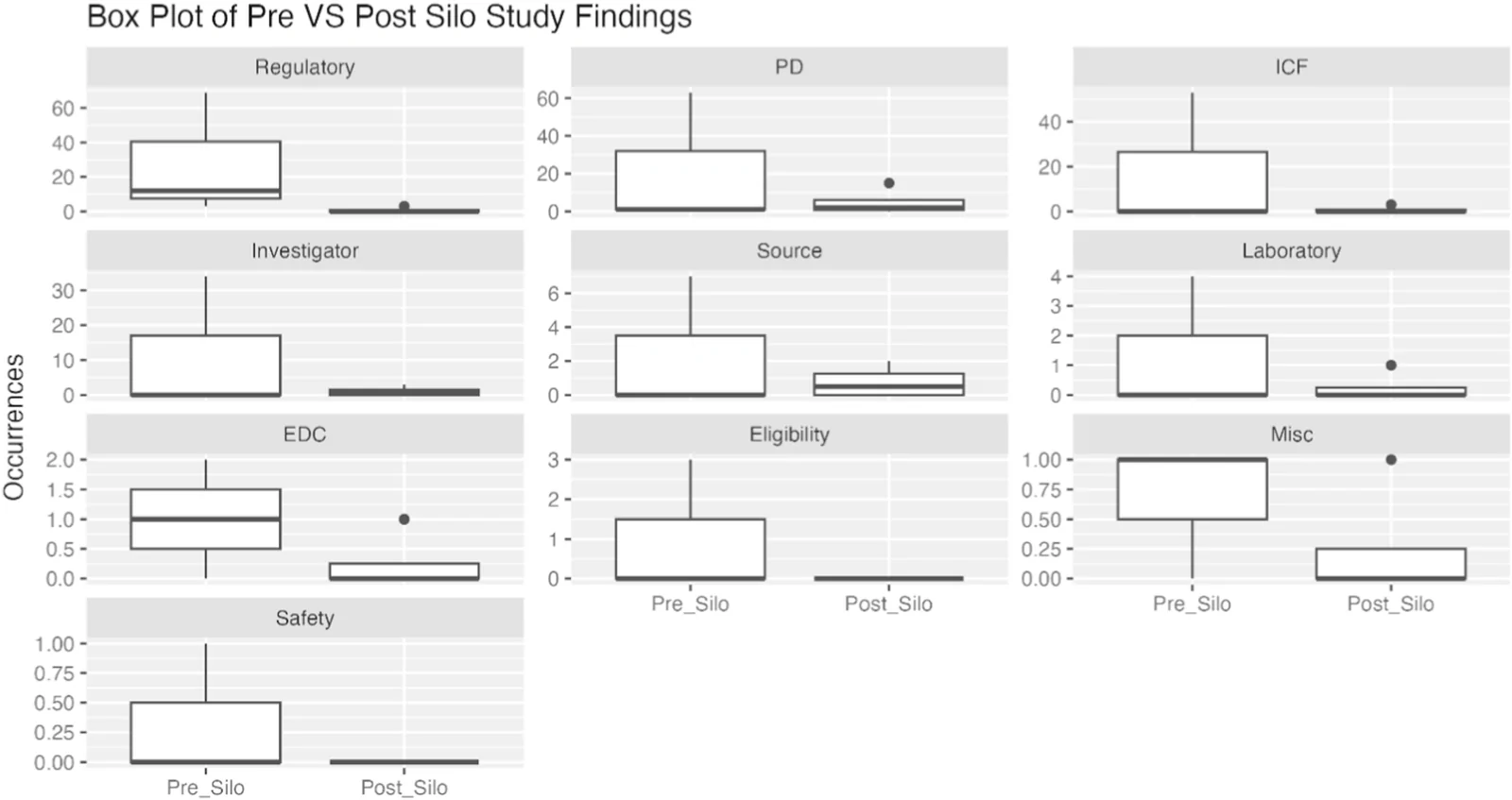
“Box plot of pre VS post silo study findings”.

## 5 Discussion

Post-implementation of the restructure of our Unit, we evaluated whether the creation of a QARS role improved study conduct. We saw a reduction in the total number of findings across almost all categories after the silo. The greatest difference was in our site's Regulatory, ICF, Investigator, and Protocol Deviation (in prevention studies) categories.

The creation of a QARS role, with duties explicitly carved out for internal regulatory and study conduct oversight, has improved the overall quality of studies conducted in the IDRU. Internal monitoring would not be possible without the silo of research duties. The internal monitoring takes place before the sponsored monitors arrive on-site, which allows the study team to resolve internal findings before the sponsored monitors identify them. In our previous framework, where one coordinator was responsible for every aspect of their trial, they would not be able to view the study data/documents objectively and see their own gaps in knowledge. The QARS individual can monitor study data and source documents for compliance purposes from an objective point of view, can identify common gaps in knowledge, teach the coordinators on these points, and can develop and implement CAPAs to prevent recurrences. The QARS individual also improves communication with the investigators, by outlining their pending action items and supporting them in their resolution. The QARS individual has become an integral part of the research unit and serves as a main contact for all study team members to reach out to for regulatory and compliance support.

Our results suggest that the creation of a QARS role may be particularly beneficial to research units operating within layered regulatory frameworks that span international, national, and institutional policies. In this setting, the QARS role functions as an operational bridge between regulatory requirements and day-to-day study conduct. This structure helps translate regulatory expectations into routine research practices while supporting participant protection, data integrity, and study quality. Our team made an effort to increase the generalizability of this pilot study by dividing study findings by trial type (e.g. prevention and treatment), so that other research units could evaluate the effectiveness of this structure on their research portfolio.

There were a few notable limitations to our data set. One major limitation is that the data we collected was not normally distributed and was non-paired, which limited our ability to perform statistical analyses. Another limitation was inconsistencies in different sponsors’ categorization of findings in their monitoring reports, so to maintain consistency we recategorized their findings into the audit toolkit categories. Further research is needed for harmonious guidelines for the categorization of findings to maintain consistency across sponsors, indications, and study design type.

## 6 Conclusion

In September 2022, our site restructured the IDRU through a pilot program. The separation of duties and creation of a QARS role, a Regulatory and Quality Assurance team (RQA), and incorporation of Process Improvement components highlighted the success of this pilot.

Moving to a siloed research unit has increased productivity, boosted enrollment, improved the quality of research conduct, and maintains a consistently high standard for all research in our division. A major component of the RQA role has been the development of Quality Assurance monitoring workflows that streamline the internal monitoring process, help keep it consistent across studies, and aid in the communication of findings to the study team. The UC Davis IDRU can provide these workflows and other resources to assist research sites in developing a sustainable clinical research program that aims to improve research quality.

## CRediT authorship contribution statement

**Elizabeth E. Robison:** Writing – review & editing, Writing – original draft, Software, Methodology, Formal analysis, Data curation. **Devon M. Estes:** Formal analysis, Data curation. **Jaskaran S. Halait:** Software, Formal analysis. **Katelyn L. Trigg:** Writing – review & editing, Writing – original draft, Supervision, Project administration, Conceptualization.

## Consent for publication

Not applicable.

## Availability of data and materials

The datasets used and/or analyzed during the current study are available from the corresponding author on reasonable request.

## Funding

The authors declare that there was no funding supporting this project.

## Declaration of competing interest

The authors declare that they have no known competing financial interests or personal relationships that could have appeared to influence the work reported in this paper.

## Data Availability

Data will be made available on request.
